# Comparative Analysis of Aducanumab, Zagotenemab and Pioglitazone as Targeted Treatment Strategies for Alzheimer’s Disease

**DOI:** 10.14336/AD.2021.0719

**Published:** 2021-12-01

**Authors:** Morteza Abyadeh, Vivek Gupta, Veer Gupta, Nitin Chitranshi, Yunqi Wu, Ardeshir Amirkhani, Anna Meyfour, Samran Sheriff, Ting Shen, Kunal Dhiman, Ghasem H. Salekdeh, Paul A. Haynes, Stuart L. Graham, Mehdi Mirzaei

**Affiliations:** ^1^Cell Science Research Center, Department of Molecular Systems Biology, Royan Institute for Stem Cell Biology and Technology, ACECR, Tehran, Iran.; ^2^Department of Clinical Medicine, Macquarie University, Macquarie Park, NSW, Australia.; ^3^School of Medicine, Deakin University, VIC, Australia.; ^4^Australian Proteome Analysis Facility, Macquarie University, Macquarie Park, NSW, Australia.; ^5^Basic and Molecular Epidemiology of Gastrointestinal Disorders Research Center, Research institute for Gastroenterology and Liver Diseases, Shahid Beheshti University of Medical Sciences, Tehran, Iran.; ^6^Department of Molecular Sciences, Macquarie University, Macquarie Park, NSW, Australia

**Keywords:** Alzheimer’s disease, Aducanumab, Zagotenemab, Pioglitazone

## Abstract

Alzheimer’s disease (AD) is the leading cause of dementia that has remained a major medical, sociocultural and economical challenge globally. Previously developed treatments like anticholinesterase inhibitors (AChEIs) and N-methyl-D-aspartate receptor (NMDAR) antagonists only provide short-term symptomatic improvement and do not prevent progression. Repeated setbacks and failures over the past 25 years in AD clinical trials have hindered efforts to develop effective AD treatments. Fortunately, Aducanumab, a specific anti-amyloid β antibody, has shown promising clinical results and was recently approved by the Food and Drug Administration (FDA) through an accelerated approval pathway. This has raised hopes for AD patients; however post-approval trials are necessary to estimate the true scope of its clinical benefits. We have reviewed several AD clinical studies and summarized the experience to date with Aducanumab and two other potential AD drugs including Zagotenemab (an anti-tau antibody) and Pioglitazone (nuclear Peroxisome-Proliferator Activated Receptor γ (PPARγ) agonist). These have shown mixed results so far and the next few years will be critical to elucidate and interpret their broad long-term protective effects. A concerted effort is required to understand and strengthen the translation of pre-clinical findings from these drugs to routine clinical practice.

Alzheimer’s disease (AD) is the most common cause of dementia; it’s also the sixth leading cause of death amongst elderly population. The disease is typically characterized by initial memory and learning impairment followed by cognitive dysfunction which may lead to behavioral, speech, visuospatial and the motor disturbances [1-4]. It is estimated that currently over 50 million people suffer from dementia with combined annual cost of the disease approximated at US $1 trillion. The number of dementia cases is expected to grow exponentially and cross 150 million mark by 2050 globally [5-7]. This has led to severe economic burden for patient families and public health sector. AD accounts for up to 75 % of all dementia cases and as such the disease requires an effective early diagnosis which has been alluding the researchers.

Although the exact mechanism underlying AD pathogenesis is being investigated, two major neuropathological hallmarks of AD are senile plaques, which are formed by extracellular accumulation of amyloid β (Aβ40/42) fibrils, and intracellular neurofibrillary tangles (NFT) which contain aberrantly hyperphosphorylated tau as paired helical filaments (PHFs). The accumulation of these misfolded proteins is also associated with inflammatory response, astrogliosis and microglial cell activation [8-11]. These two pathological hallmarks and their downstream events are the main targets of drug discovery studies.

Amyloid plaques consist of 39- to 42-amino acid β-amyloid peptides derived from a large single-pass transmembrane amyloid precursor protein (APP) [12, 13]. APP is initially cleaved by BACE1 enzyme and generates C99 fragment and a soluble APPβ region. In the subsequent step C99 undergoes a cleavage by γ-secretase enzyme and produces the neurotoxic forms of Aβ [14-17]. The imbalance in production, assembly, and clearance of Aβ has been suggested as key pathological mechanism in AD. The clearance of Aβ may be affected by several reasons including environmental and genetic factors such as apolipoprotein E polymorphism (Apo E2/3/4). Apo E4 isoform in particular has been showed to be associated with decreased Aβ catabolism and increased risk of AD [6].

According to the amyloid hypothesis, amyloid plaques may promote inflammation and oxidative damage which contribute to molecular changes implicated in neurodegenerative processes [18, 19]. Moreover, Aβ pathology has been shown to co-exist with and reinforce tau protein pathology [20, 21]. Tau is a major micro-tubule associated protein that has been implicated in AD neuropathology. It plays key roles in axonal transport, neurite outgrowth, synaptic plasticity and maintaining neuronal polarity through stabilizing of the microtubular cytoskeleton. Tau is highly soluble protein that can undergo phosphorylation at various amino acid residues. The protein phosphorylation is regulated by several kinases and phosphatases, including glycogen synthase kinase-3 (GSK-3), casein kinase 1 (CK1), cyclin-dependent kinase 5 (cdk5), 5′ adenosine monophosphate-activated protein kinase (AMPK), microtubule affinity-regulating kinases (MARKs), protein phosphatase-1, 2A, and 5 (PP1, PP2A, and PP5)[22-24]. Abnormal hyperphosphorylation of tau (p-tau) affects its affinity to bind microtubules and leads to micro-tubular dysfunction, the enhanced cytosolic level of p-tau subsequently form NFTs [25, 26] which are associated with axonal degeneration, synaptic dysfunction and neuronal loss in AD [6, 21].

In addition to the roles of Aβ and tau protein, there is a growing body of evidence indicating a link between metabolic disorders and AD pathology. Several studies suggest a function of insulin resistance in pathophysiology of AD [27, 28]. The association of Apo E gene polymorphism; which has a regulatory role in lipid metabolism [29], with both the risk of developing AD and type 2 diabetes mellitus (T2DM) supports this line of research [6, 30]. Diabetes is a risk factor for the development of dementia and the disease was shown to enhance cognitive decline in APOE2/ *APOE3*, but not *APOE4* genotypes. Peroxisome proliferator-activated receptor gamma (PPAR-γ) agonists modulate glucose and lipid metabolism and are approved by FDA for the management of hyperglycemia and lipid disorders. Recently, the role of PPARϒ in neurodegeneration and cognitive impairment is gaining ground. The drug is shown to modulate inflammatory response and reduce amyloid burden and improve behavioural deficits in animal models. The drug has also shown beneficial effects on cognition and memory in AD patients in clinical trials [31].

Previously FDA approved treatments such as anticholinesterase inhibitors (AChEIs) (donepezil, galantamine and rivastigmine) and N-methyl-D-aspartate receptor (NMDAR) antagonist (memantine), provide modest symptomatic benefits but do not affect disease progression. Therefore, development of new treatment strategies targeting different aspects of the disease pathology to slow its progression is a global priority and several potential therapies have entered phase III clinical trials. We have aimed to summarize recent data on three leading AD treatment strategies including a newly FDA approved anti-amyloid β antibody (Aducanumab)), an anti-tau antibody (Zagotenemab) and a nuclear Peroxisome-Proliferator Activated Receptor γ (PPARγ) agonist (Pioglitazone) from pre-clinical to clinical studies (www.clinicaltrials.gov, accessed November 22, 2020; Table 1).

## ADUCANUMAB

Some elderly individuals show increased resistance to development of Alzheimer’s disease pathology or do not show significant cognitive declines during aging, which suggests their immune system successfully hold Alzheimer’s disease at bay. This was the driving idea behind the Aducanumab (BIIB037) discovery by scientists at Neurimmune through a reverse translational medical approach. Neurimmune in 2007 licensed Aducanumab to Biogen, who then developed it as a potential therapeutic product.

**Table 1:** Clinical Trials of Aducanumab, Zagotenemab and Pioglitazone for Alzheimer’s disease.

NCT number	Start year	Completion date	Phase	Sample size^*^	Participants	Age (years)	Dose	Available outcomes
**Aducanumab**								
**NCT01397539**	2011	Completed in 2013	1	53	Mild to moderate AD	55-85	03, 1, 3, 10, 20, 30, 60 mg/kg SD IV	Abnormal ARIA-E in 100% of 60 mg/kg but completely resolved by weeks 8-15
**NCT01677572**	2012	Terminated in 2019	1	197	Prodromal or mild AD	50-90	1, 3, 6, 10 mg/kg IV every 4 weeks	Abnormal ARIA in 2%, 13%, 37%, 47% of four dose groups - Slowing decline in CDR (10 mg/kg) and MMSE (3, 10 mg/kg)
**NCT02434718**	2015	Completed in 2016	1	21	Mild to moderate AD	55-85	Single and multiple of low/mild or high dose IV	-
**NCT02782975**	2016	Completed in 2017	1	28	Healthy participants	18-55	Single (?) dose SC and IV	-
**NCT02477800**	2015	Terminated in 2019	3	1647	Early AD	50-85	Low or high dose/monthly IV	Terminated based on futility analysis done and not based on safety concerns
**NCT02484547**	2015	Terminated in 2019	3	1638	Early AD	50-85	Low or high dose/monthly IV	Terminated based on futility analysis done and not based on safety concerns
**NCT03639987**	2018	Terminated in 2019	2	52	MCI-AD or mild AD	50-85	Every 4 weeks for up to Week 52 IV	Discontinued based on futility analysis conducted on two above phase 3 trials, not based on safety concerns
**NCT04241068**	2020	Expected to be finished in 2023	3	2400	Participated in NCT01677572, NCT02477800, NCT02484547, NCT03639987	Child, Adult, Older Adult	10mg/kg every 4 weeks for up to 100 weeks	-
**Zagotenemab**								
**NCT02754830**	2016	Completed in 2018	1	110	Healthy, MCI-AD, Mild to moderate AD	30≤	Single (?) dose IV or SC	-
**NCT03019536**	2017	Completed in 2019	1	24	MCI-AD, Mild to moderate AD	50≤	Multiple (?) doses IV	-
**NCT03518073**	2018	Expected to be finished in 2021	2	285	Early Symptomatic AD	60-85	Two different (?) doses IV	-
**Pioglitazone**								
**NCT00982202**	2002	Completed in 2005	2	25	Nondiabetic patients with AD	50≤	1 to 3 tablets (15mg-45mg) daily	Peripheral edema in treated group 28.6% compared to 0% in placebo group - No efficacy was demonstrated.
**NCT01456117**	2011	Completed in 2012	1	61	Healthy elderly participants	55-83	3 different (?) doses daily up to 14 days	-
**NCT01931566**	2013	Terminated in 2018	3	3494	Low and high risk of MCI-AD	65-83	1 sustained release tablet (0.8 mg) daily	Discontinued due to lack of efficacy of the drug; no safety concern
**NCT02284906**	2015	Terminated in 2019	3	40	Participated in NCT01931566 and high risk of MCI-AD	65≤	1 tablet (0.8 mg) daily	Discontinued due to lack of efficacy of the drug; no safety concern

AD, Alzheimer’s disease; SD, Single dose; IV, Intravenous; SC, Sub-cutaneous; MCI, Mild cognitive impairment; MCI-AD, Mild cognitive impairment due to Alzheimer disease (?) Dose is not reported;

* Information is obtained from clinicaltrials.gov.

**Figure 1.**
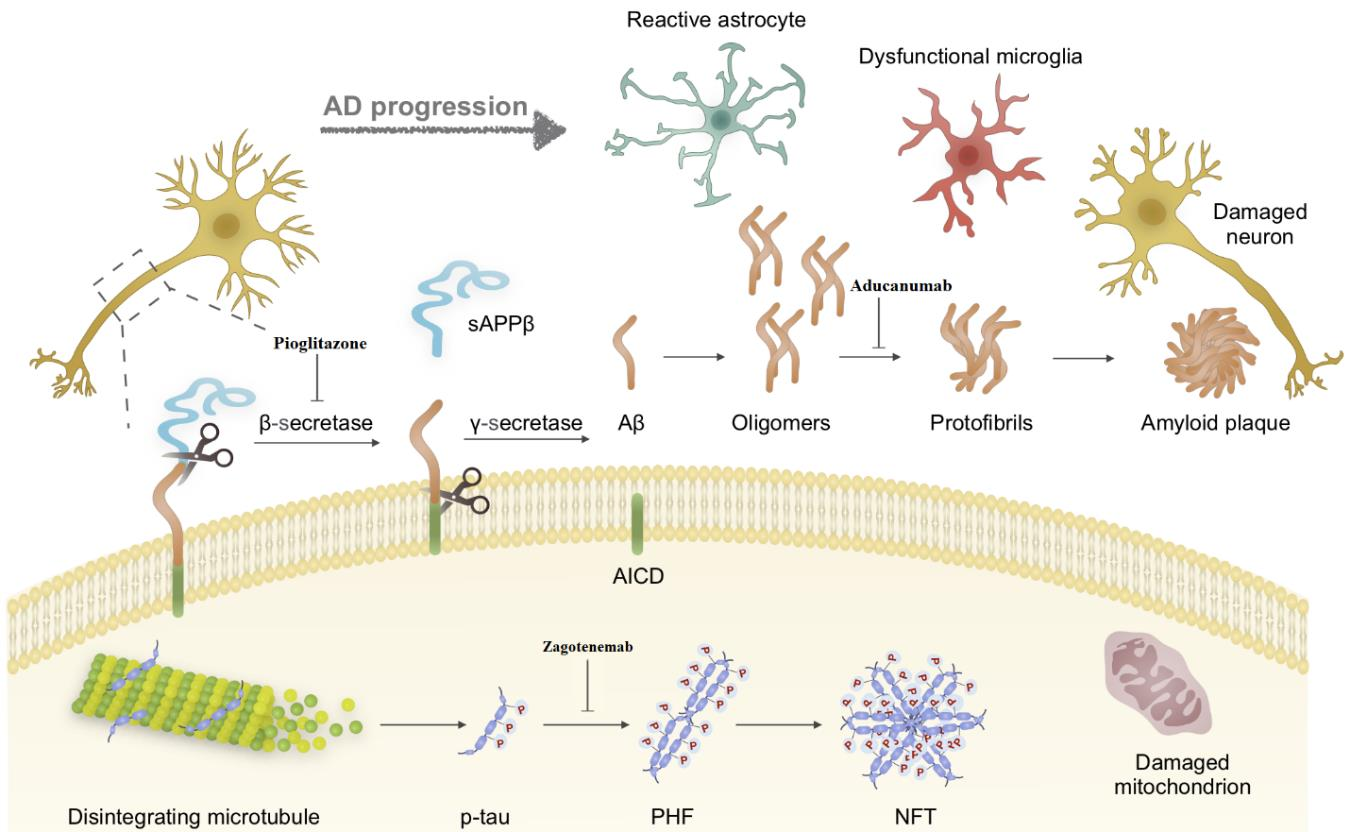
**Alzheimer pathogenesis and the main targets of disease-modifying agents.** Aβ is produced from APP via the proteolytic functions of β- and γ-secretase, which aggregates and form amyloid plaques; aberrant phosphorylation and conformational change of Tau decreases its affinity to bind microtubules and triggers aggregation in a concentration dependent manner promoting NFT formation. These processes ultimately lead to AD pathogenesis including neuronal damage, astrocyte activation, microglia dysfunction and mitochondrial damage. Aducanumab has a higher affinity for Aβ aggregates than monomers, Zagotenemab binds to a toxic conformation of Tau protein, and Pioglitazone affects the β- secretase activity by reducing its gene expression. PHF, paired helical filaments; NFT, intracellular neurofibrillary tangles; AICD, amyloid precursor protein intracellular domain; BACE, β- secretase; sAPPβ, soluble amyloid precursor protein- β.

Aducanumab is a human immunoglobulin G1 monoclonal antibody (originally known as BIIB037), that recognizes aggregated forms of amyloid beta (Aβ) including soluble oligomers and insoluble fibrils (Fig. 1). It was obtained from a B-cell library collected from healthy elderly subjects who were cognitively normal [32, 33]. Aducanumab binds to a linear epitope formed by amino acids 3-7 of the Aβ peptide and has a higher affinity for fibrillar aggregates than monomers, based on weak monovalent affinity, fast binding kinetics and strong avidity for epitope-rich aggregates [34, 35]. BIIB037 was reported to be able to cross the blood-brain barrier and react with Aβ aggregates with > 10,000-fold more selectivity than monomers. Peripheral administration of murine analogs of BIIB037 reduced amyloid load from plaque-bearing transgenic mouse brains [36, 37].

Calcium homeostasis plays a key role in correct neuronal function. Calcium overload in neurites (neuronal dendrites and axons) as a consequence of senile plaque deposition has been reported to be correlated with structural and functional disruption of neurons in APP mice [38, 39]. In this regard, a single topical application of Aducanumab was shown to rapidly remove amyloid plaques in 18 months old Tg2576 mouse. Unlike the topical application, chronic systemic application of Aducanumab for 6 months did not affect existing plaques but restored calcium homeostasis in 21 months old Tg2576 mice, which might be due to the resistance of senile plaques with significant density in older mice [40]. It was suggested that Aducanumab might target the soluble species of Aβ, not Aβ aggregates, and improve the cognitive decline by ameliorating calcium overload and restoring neuronal function [41]. Furthermore, a recent proteomics study showed that chronic systemic administration of Aducanumab restored the impaired calcium homeostasis in a mouse model of AD (10-month-old tgAPPPS1-21 mice). Analyzing the proteomic changes in senile plaques and surrounding tissue showed that Aducanumab administration caused an increase in abundance of proteins involved in phagocytosis, metabolism, and neuronal and axonal regeneration, and a decrease in abundance of proteins involved in stress and Aβ toxicity [42].

In 2011, a phase 1a clinical trial (NCT0139 7539) was initiated to evaluate the safety and tolerability of intravenous administration of Aducanumab with different doses (0.3, 1, 3, 10, 20, 30, and 60 mg/kg) to determine the maximum tolerated dose (MTD) in 53 patients with mild to moderate AD, and also to assess the pharmacokinetics (PK) and immunogenicity of this mAb. In 2016, outcome of this clinical trial as the first study of Aducanumab intervention in humans was published by Ferrero and colleagues. Outcomes from this study with 39 patients (although 53 patients are reported in ClinicalTrials.gov) from 3 sites in the United States, showed acceptable safety and tolerability of doses ≤ 30 mg/kg with no serious adverse events. However, abnormal ARIA-E (Alzheimer's Related Imaging Abnormality-Edema) were observed in all three patients that received the highest dose of 60 mg/kg, and the symptoms were resolved after 8-15 weeks [43]. Finally, it was established that doses ≤10 mg/kg were better tolerated and as such selected for subsequent clinical trials.

Based on these results, in 2012, the phase Ib clinical trial (NCT01677572) initiated by Biogen used doses ≤ 10 mg/kg (1, 3, 6 or 10 mg kg^-1^) in 197 participants with prodromal or mild AD to assess the safety and tolerability of these concentrations further based on phase Ia observations; examine the efficacy of the mAb on reducing the cerebral Aβ plaque content as evaluated by florbetapir-fluorine-18 (18F-AV-45F-AV-45) Positron Emission Tomography (PET) imaging; and determine the serum concentrations of Aducanumab and serum anti-Aducanumab antibodies, in order to determine the optimum dose for phase II. This study was terminated in 2019 due to futility analysis performed on Phase 3 trials. Published results of this phase Ib trial in 2016 by Sevigny and colleagues showed a dose- and time-dependent brain Aβ reduction (measured by PET) in patients with prodromal or mild Alzheimer's disease that received monthly Aducanumab with different doses (1, 3, 6 or 10 mg kg^-1^) for one year. Furthermore, a dose-dependent decline in MMSE (Mini Mental Status Examination) and CDR- SB scores (Clinical Dementia Rating Scale Sum of Boxes) was observed and after 12 months, patients that received 10 mg/kg dose were negative on amyloid PET scans. In this study, initially 165 patients (197 in ClinicalTrials.gov) from 33 sites in the United States were recruited. Of those, 125 patients completed the study, while 20 patients discontinued treatment because of adverse effects which were mostly amyloid-related imaging abnormalities (ARIA), headache, urinary tract infection, and upper respiratory tract infection. However, based on the published results, these adverse effects were not significantly different between placebo and treated groups, except for ARIA and headache which were increased in treated groups. A dose dependent response could be observed for ARIA (47% for 10 mg kg^-1^); moreover, the ARIA, especially ARIA-E, was more common in APOE ε4 carriers than APOE ε4 non carriers in treated groups (55% versus 17% in 10 mg kg-1 group). In this study, anti-Aducanumab antibodies with minimal titers were observed only in 3% of Aducanumab treated patients, which had no clear impact on Aducanumab pharmacokinetics [44, 45].

There are two other phase 1 clinical trials, one of which commenced in 2015 (NCT02434718) to assess the safety, tolerability and serum PK of single and multiple IV infusions of Aducanumab in 21 Japanese participants with mild to moderate AD. The other commenced in 2016 (NCT02782975) to compare the absolute bioavailability and PK of a single, fixed sub-cutaneous (SC) dose of Aducanumab with a single, weight-based IV dose, and also assess the safety and tolerability of Aducanumab administration through these two routes in 28 healthy participants. Both of these studies lasted for a year; however, dose information and results are not yet available.

In 2018, a phase II clinical trial known as EVOLVE (NCT03639987) started, to evaluate the safety, tolerability, PK, and immunogenicity of continuing Aducanumab dosing (doses are not available yet) in asymptomatic ARIA in 52 patients with MCI (Mild Cognitive Impairment) due to AD or with mild AD. This study terminated because of futility analysis performed on phase 3 trials and results are not yet available.

Based on the promising interim analysis of the phase 1b trial, in 2015, two 78 weeklong, identically designed phase III clinical trials were initiated by Biogen; these were known as ENGAGE (NCT02477800), with 1647 participants, and EMERGE (NCT02484547), with 1638 early AD patients. The primary outcome measure of these trials was to investigate the effectiveness of monthly administration of Aducanumab in slowing cognitive decline and functional impairment as assessed by changes in the CDR-SB score. The secondary outcome measures were changes in clinical progression assessed by MMSE, ADAS-Cog 13, and ADCS-ADL-MCI. These trials had three groups, cases that received low doses of Aducanumab (3 mg/kg for APOE ε4 carriers, 6 mg/kg for non- carriers), high dose of Aducanumab (10 mg/kg), or placebo (https://investors.biogen.com; EMERGE and ENGAGE Topline Results).

In March 2019, Biogen announced the discontinuation of aducanumab clinical development temporarily since an interim analysis of phase III trial outcomes showed that Aducanumab treatment was not able to reproduce its positive results from initial trials that demonstrated slowing cognitive decline. However, 7 months later on October 2019, Biogen claimed that their initial analysis was based on less accurate statistical approach and subsequent analysis of a larger data set revealed that Aducanumab in the EMERGE trial was indeed able to meet its primary endpoint [37, 46]. Specifically, in patients that received a high dose (10 mg/kg), after 26 weeks a significant reduction in cognitive decline was observed based on CDR-Sum of Boxes and also secondary cognitive endpoints including MMSE, ADAS-Cog, and ADCS-ADL-MCI tests. Furthermore, behavioral disruptions including anxiety, agitation, aggression, and depression that were assessed by Neuropsychiatric Inventory - 10-item version (NPI-10) showed 87% reduction compared to placebo group. However, the changes did not reach statistical significance for the lower dose group [37, 46].

Unlike the EMERGE results, the ENGAGE results did not meet its primary endpoint, although a subgroup analysis showed that patients who received 14 doses of 10 mg/kg showed a reduction in CDR-SB-measured cognitive decline compared to EMERGE patients. Moreover, results from ENGAGE and EMERGE sub-studies that investigated cerebrospinal fluid (CSF) biomarkers showed that Aducanumab significantly reduced CSF p-tau in a dose dependent manner, and also a dose depended reduction was observed for CSF t-tau (total tau) that is a biomarker of neurodegeneration. Moreover, decreases in temporal, medial temporal and frontal composites of standard uptake value ratios (SUVR) on tau PET were observed. Furthermore, a dose dependent reduction was observed in amyloid PET SUVR. Collectively, these results demonstrated the therapeutic benefits of Aducanumab (https://investors.biogen.com; EMERGE and ENGAGE Topline Results). ARIA was the common side-effect of Aducanumab which was notable in clinical results, but it was transient and manageable using a titration schedule and safety monitoring with MRI. Based on these results, in January 2020 Biogen invited about 2400 patients that had previously participated in ENGAGE and EMERGE trials to participate in another phase III trial (NCT04241068), to assess the long-term safety and tolerability of this mAb after a wash-out period occurred by termination of previous trials (EMERGE and ENGAGE), and in July 2020, Biogen submitted a New Drug Application to the Food and Drug Administration (FDA). In June 2021 FDA approved Aducanumab (marketed as Aduhelm) as a drug for AD through accelerated approval pathway, which is used when drug is the first available treatment or shows advantages over existing treatments for life-threatening illness and is likely to predict clinical benefit based on a surrogate endpoint, however Biogen is still required to conduct post-approval trial (known as phase 4 confirmatory trial) to verify the anticipated clinical benefit. Now aducanumab is the first approved drug for AD in nearly 20 years and also the first therapy which has provided evidence that clearing amyloid beta caused better clinical outcomes.

## ZAGOTENEMAB

Zagotenemab (LY3303560) is an anti-tau antibody that selectively binds and neutralizes Tau deposits in the brain. This antibody is derived from a monoclonal antibody known as MCI-1 and is being developed by Eli Lilly and Company as a potential drug for AD patients. MCI-1 is a conformation-selective anti-Tau antibody that binds to an early pathological form of Tau conformation which is soluble and forms before the assembly of PHF (Figure 1) [47]. Thus, the MC-1 epitope is one of the earliest events in the development of AD and offers a promising approach for early treatment. MC-1 was introduced in 1997 by Davies and colleagues [48]. Another conformation-dependent anti-Tau antibody had previously been developed by this group, known as Alz-50 [49], but it showed cross reactivity with FAC1 (Fetal Alz-50-Reactive Clone 1), a protein with a functional role during neuronal development and degeneration [48, 50]. MC-1 is similar but not identical to Alz-50, and does not react with FAC1 [48].

In 2011, Chai and colleagues showed that passive immunotherapy for Tau with intra-peritoneal injection of MC-1 in two transgenic models of Tau pathogenesis, JNPL3 and P301S, attenuated levels of hyper-phosphorylated insoluble Tau protein and neurofibrillary tangles in the in the forebrain, and also notably delayed the onset of motor dysfunction and weight loss [51]. Furthermore, Vitale and colleagues used the same mice model (JNPL3) and showed that direct hippocampal injection of single-chain variable fragment of MC1 (scFv-MC1), in an AAV delivery system, effectively induced astrocyte activity and reduced soluble, oligomeric and insoluble tau species even in areas distant from the injection site, including the cortex and hindbrain [52]. Another study by this group on the same mice models showed that a single intramuscular (IM) injection of the previously used vectorized scFv derived from MC-1 stimulated long term production of anti-tau scFvMC1, and significantly decreased insoluble and soluble tau in different brain regions. In addition, this study highlighted the key role of microglia in clearance of scFv-tau [53]. The role of microglia in MC-1 mediated tau clearance was also assessed by Luo and colleagues, who used the P301S mice model and showed that MC-1 accelerates the uptake and clearance of pathological tau species by microglia in an Fc-dependent manner [54].

There are three approaches in Tau immunotherapy based on targeting total tau, tau phosphorylation, or conformational epitopes, each of which shows different efficacy and safety. In this regard, conformational based Tau targeting with MC-1 showed improved therapeutic features over other approaches. In a comparison between MC-1 and DA31 (a high affinity tau sequence antibody) in mutant P301L mice, MC-1 caused a significant reduction in total Tau and insoluble Tau in forebrains of P301L Mice, and was more effective than DA31 in reducing the rate of tau pathology development, which highlighted the importance of specificity rather than affinity in therapeutic applications [55]. A subsequent study by Hayashi and colleagues in 2017 indicated the superiority of MC-1 over other Tau antibodies in neutralization of transmissible Tau and attenuation of Tau pathology both *in vitro* and *in vivo.* Mc-1 was found to be superior to PHF-1 (against phosphorylated Tau on serine 396 and 404), AT-8 (against phosphorylated Tau) and DA-9 (against all Tau forms) [56]. Furthermore, Alam and colleagues in 2017 developed a humanized anti-Tau antibody derived from MCI-1 (LY3303560), and in a preclinical study on rat and monkey showed that LY3303560 binds to a epitope located in the N-terminal region of Tau protein, and has a higher affinity for Tau aggregates than for monomers [57].

In early 2016, a 16-week phase I clinical trial (NCT02754830) was started, to assess the safety and tolerability of intravenous administered single doses of Zagotenemab in 110 patients with MCI due to AD or mild-to-moderate AD, along with healthy volunteers. The primary outcome measure in this study was number of individuals with one or more SAEs (Serious Adverse Events), secondary outcome measures included: serum and cerebrospinal fluid AUC [0-∞] (Area Under the Concentration versus Time Curve from Time 0 to Infinity), C*max* (Maximum Drug Concentration), and mean change from baseline in QTc (QT/QT Corrected). This study finished in 2018 but details are not yet published. In 2017, a second phase I clinical trial (NCT03019536) was initiated, to examine the safety of repeated doses of injected Zagotenemab for 25 weeks in 24 MCI or mild-to-moderate AD patients. The primary outcome measure was SAEs, and secondary outcome measures were serum C*max* and AUC (Area Under the Concentration Versus Time Curve). This study finished in 2019 but results are yet to be published. However, the efficacy of this drug was only significant for early stages of AD, since in phase II clinical trial only early symptomatic AD patients were recruited; the phase II clinical trial (NCT03518073) started in 2018 to examine the safety, tolerability and efficacy of Zagotenemab in 285 in patients with early symptomatic AD patients is ongoing and participants will receive intravenously one of two different doses of Zagotenemab or a placebo. The primary measured outcome is change from baseline on the iADRS, and the secondary outcomes include change from baseline on the ADAS-Cog13, ADCS-iADL, CDR-SB, and MMSE scores. This study is expected to be finished in 2021.

## PIOGLITAZONE

The thiazolidinedione derivative known as Pioglitazone is a peroxisome proliferator-activated receptor gamma (PPAR-γ) agonist developed by the Takeda pharmaceutical company and approved by FDA in 1999 for management of type 2 diabetes. PPAR-γ, also known as NR1C3, is a type of nuclear receptor that forms a heterodimer with Retinoid X Receptors (RXRs) and modulates the transcription of several genes responsible for lipid and glucose metabolism and inflammation. Pioglitazone binds to PPAR-γ and consequently reduce hyperglycemia, hyperinsulinemia, hyper-triglyceridemia, and inflammation [58-61].

Cumulative epidemiological studies indicate the decreased risk of AD in individuals that are subjected to a long-term treatment with non-steroidal anti-inflammatory drugs (NSAIDs) [62]. Reducing the expression of proinflammatory genes by activating the PPARγ is considered to contribute to the NSAIDs protective features against AD [63-66], and therefore Pioglitazone has gained a great deal of attention as a potential treatment for AD. In this regard, Yan and colleagues in 2003 reported that oral administration of 20 mg/kg/day pioglitazone for 4 months modestly reduced SDS-soluble Aβ levels but had no significant effects on microglial activation and amyloid plaque burden in the brain of APP-expressing Tg2576 transgenic mice [67]. However, two years later, Heneka and colleagues demonstrated that oral administration of a higher dose of Pioglitazone (40 mg/kg/day) for 7 days significantly decreased the expression of BACE 1, glial inflammation, and soluble and deposited Aβ1-42 levels in the brain of 10-month-old APPV717I-overexpressing mice [68]. This inconsistency between these two results is probably due to the limited amount of Pioglitazone that can cross the blood-brain barrier (about 18%) when given orally [68, 69]. Crenshaw and colleagues subsequently reported that treatment with Pioglitazone at a low dose (0.08 mg/kg/day), but not a higher dose (0.32 mg/kg/day), significantly increased the functional connectivity between the CA1 region, a region with function in memory which is affected in early AD, and the hypothalamus and the ventral thalamus in Wistar rats. This study suggests that Pioglitazone improves neuronal and/or cerebrovascular function at lower doses compared to those applied for T2DM treatment [70]. Moreover, in 2006, Sastre and colleagues also showed that PPAR-γ activation by Pioglitazone reduced BACE1 gene promoter activity by binding to a PPAR-γ responsive element (PPRE) located in the BACE1 gene promoter [71]. Furthermore, Mandrekar-Colucci and colleagues reported that PPAR-γ activation by 9 days of administration of Pioglitazone in APP/PS1 mice enhanced the expression of Apolipoprotein E (ApoE), ABCA1 and Liver X receptors, and led to clearance of soluble Aβ, Pioglitazone administration also affected the polarization of microglial cells, which changed from a proinflammatory M1 state into an anti-inflammatory M2 state with enhanced phagocytosis of deposited Aβ [72]. Another study also showed that a low dose of Pioglitazone (2 mg/kg/day) increased the expression of low-density lipoprotein receptor-related protein 1 (LRP1), a large endocytic receptor that is critically involved in brain Aβ clearance, and reduced Aβ in the hippocampus of a mouse model of sporadic AD which consequently ameliorated the learning and memory impairment. The higher dose (5 mg/kg/day) failed to achieve these results. These doses, 2 and 5 mg/day in mice, could be approximately equivalent to 10 and 26 mg/day in humans [73-76].

Pioglitazone also showed efficacy in targeting several changes in AD that occur before the appearance of senile plaques and NFTs [77]. Collapsin response mediator protein 2 (CRMP2) is a brain protein with a key role in axonal guidance, correct dendritic organization, and microtubule stability. In AD, abnormal phosphorylation of CRMP impairs its functions, and this phosphorylated form is also detected in NFTs [78, 79]. It has been reported that treatment with Pioglitazone normalized the altered CRMP2 phosphorylation level, p35 protein level, impaired motor coordination ability and long-term depression (LTD) at the pre-Aβ accumulation stage in APP/PS1 mice model of AD [77].

The efficacy of Pioglitazone can be enhanced by combination therapy, as it was reported that combined treatment in AD model mice with Pioglitazone and Fenofibrate, a fibric acid derivative which is used to treat abnormal blood lipid levels, for 21 days in a mice model of AD showed better results than monotherapy. This included recovering the reduced levels of Wnt, β-catenin and PPARα/ β, and increased levels of α- and β-secretase, and also significantly improving the impaired memory and cognition, as indicated by behavioral tests [80].

Based on observed safety of 15-45 mg/day Pioglitazone in type 2 diabetes clinical trials, in 2002, an 18-month phase II clinical trial (NCT 00982202) to evaluate the safety and tolerability of pioglitazone in 29 nondiabetic AD patients was started by the National Institute on Aging (NIA) in collaboration with Takeda Pharmaceuticals North America. The clinical measures of cognition, daily function, and behavior were also assessed as the secondary outcomes [81]. Results of this trial showed that 25 participants finished the study and there were 4 primary withdrawals due to change in caregiver status and withdrawal of consent, not related to adverse effects. Participants were given either 1 pioglitazone tablet (15 mg), or matched placebo, daily at first and then daily dosing escalated by 1 tablet each week to maximum 3 tablets daily (45 mg). No statistically significant differences in the terms of education, sex, ethnicity, APOE4 status, or MMSE score at baseline were recorded between participants. Pioglitazone was well tolerated, with no notable effect on blood glucose levels, hemoglobin A_1C_ levels, or other blood chemistry or hematologic measures. Peripheral edema was the only adverse effect that was notably different between pioglitazone-treated and placebo groups, which was expected as it is a commonly recognized side-effect of Pioglitazone in clinical use. At the end, no efficacy was demonstrated on clinical outcome measures and the reasons underlying these disappointing results remain unclear. However, considering the animal studies, disease severity may play a role and further study in earlier disease stages like mild cognitive impairment may provide more promising results [81].

In 2011, another phase I clinical study (NCT01456117) was started by Takeda to evaluate the effect of daily pioglitazone administration with three different doses for 14 days on brain hemodynamics in 61 healthy elderly participants. This study was completed in 2012 but results are not published yet.

**Table 2:** Pros and cons of each drug based on both experimental and clinical studies.

Drug name	Current status	Pros	Cons
**Aducanumab**	FDA approved (June 2021)	Target Aβ aggregates with specificity and high-affinity Reduce CSF p-tau and t-tau Reduce clinical decline in cognition, daily function and behavioral symptoms in patients Restore impaired calcium homeostasis	Side effects including: ARIA, headache, brain microhemorrhage, fall, superficial siderosis and diarrhea Expensive (approximately $56,000 a year) Monthly intravenous infusion Effective for early stages of AD and might not be effective in advanced stages due to presence of senile plaques with significant density (based on mice studies; it's never been tested in people with moderate to severe disease)
**Zagotenemab**	Ongoing clinical trial phase II	Binds to an early pathological form of Tau conformation that occurs before PHF formation Higher affinity for Tau aggregates than for monomers Reduce levels of both soluble and insoluble p-tau protein and NFTs Delays the onset of motor dysfunction and weight loss	Possibly is useful only for patients with early symptomatic AD (MCI-AD) Clinical data about efficacy and safety are not available yet
**Pioglitazone**	Stopped in phase III clinical trial	Is is primarily adiabetes drug, therefore can be more beneficial for diabetic patients with AD Low side effects (only Peripheral edema) Reduce soluble Aβ levels through increasing clearance of Aβ Reduce senile plaques and glial inflammation in high doses (40mg/kg/day) Ameliorate learning and memory impairment	No significant treatment efficacy in clinical trials (possibly due to using low doses). Low BBB penetration May be only useful for patients with early symptomatic AD (MCI-AD) Has no effect on senile plaques in low doses (20 mg/kg/day)

AD, Alzheimer’s disease; ARIA, Amyloid-related imaging abnormality; MCI-AD, Mild cognitive impairment due to Alzheimer disease; PHF, Paired helical filaments; NFTs, Neurofibrillary tangles; BBB, Blood brain barrier.

Two years later in 2013, Takeda in collaboration with Zinfandel Pharmaceuticals started a 5-year phase III clinical trial (NCT 01931566) with 3494 participants from 57 investigative sites in the United States, United Kingdom, Germany, Australia, and Switzerland. The aims were to establish a new genetic test to determine if participants are at risk of developing MCI due to AD within the next five years, and also to evaluate the efficacy of daily 0.8 mg Pioglitazone administration for up to five years to delay the onset of MCI-AD in cognitively-normal people that were susceptible for developing MCI-AD. This study (known as TOMMORROW) was terminated in 2018 due to lack of Pioglitazone efficacy, although no adverse effects were observed.

In 2015, another phase III clinical trial (NCT 02284906) was started by Takeda to assess the efficacy of daily 0.8 mg Pioglitazone use for 24 months to slow cognitive decline in 40 high-risk participants who completed the previous phase III clinical trial (NCT01931566) with an adjudicated diagnosis of MCI due to AD. This study also was terminated in 2018 due to lack of Pioglitazone efficacy.

Despite a plethora of *in vitro* and *in vivo* evidence, testing for the efficacy of Pioglitazone has been fraught with failure and confusing results from clinical trials, which could be due to employing low doses of Pioglitazone (0.8mg sustained release (SR) tablets/day) compared to animal studies (2-40mg/kg/day), and also its low penetration of the blood-brain barrier (Table 2) [68]. There is an *in vivo* report on a mouse model of AD indicating the efficacy of Pioglitazone in low doses is even better than for higher doses, which suggested using Pioglitazone to treat AD at lower doses than what is used for treatment of T2DM (15-45mg/day) [76], but even these doses were higher than what was used in these clinical trials.

### Conclusion and future directions

Promising results from Aducanumab clinical trials and recent FDA approval have raised hopes for AD patients and provided a renewed attention for amyloid hypothesis. FDA has recently granted an accelerated approval for the drug use, in early but not in advanced stages of AD. However, if post-approval trials confirm the estimated clinical benefits, this will be a historic breakthrough that will potentially result in reduced burden of AD globally. Therefore, next few years will be critical for Aducanumab clinical development and further research will unravel its therapeutic applications in various types of dementia and AD stages. Whilst individually each of the aforementioned drug therapies have previously shown to illicit neuroprotective and anti-inflammatory actions, a combination therapy approach may be required clinically to address the complexity of AD and the various pathological challenges associated with the disease. Martos and colleagues in 2017 studied the impact that a combination therapy consisting of leptin and pioglitazone may offer in a 6-month-old APP/PS1 (APPswe/ PSEN1dE9) transgenic AD mouse model. They concluded that after a 2 week treatment period, combination with leptin and pioglitazone resulted in reduction of spatial memory defects (Y Maze) and brain amyloid levels relative to vehicle treated controls [82, 83]. With several new drug trials and therapies failing large phase 3 clinical trials, it highlights the complexity of AD treatment and the pathological nature of the disease. Additional trials investigating logical combinations of agents should continue to be performed in order to understand the relationships among the numerous neurodegenerative pathways. The key reason for failure of Pioglitozone trials to meet primary end-points is likely due to the use of low doses in clinical trials when compared to doses used in preclinical studies. Promising results from high dose of aducanumab in clinical trials also suggest the need for higher doses of treatment. Moreover, low brain penetration due to the presence of the blood brain barrier or existence of too much Aβ plaque or NFT also undermines the treatment’s effectiveness. Enhancing the drugs bioavailability through higher doses or employing novel delivery systems to increase brain penetration, like using nano-carriers, or initiating the trials even earlier in the course of AD could yield better results. For Zagotenemab, the main advantage is its ability to target early and specific events in AD development which potentially increases the chance of successful translation. Hopefully, clinical trials will continue to yield results that are as promising as those observed in preclinical studies in rodent and primate models.
